# Di-μ-bromido-bis­[bromido(4,4′-dimethyl-2,2′-bipyridine-κ^2^ *N*,*N*′)mercury(II)]

**DOI:** 10.1107/S1600536808032510

**Published:** 2008-10-15

**Authors:** Khadijeh Kalateh, Amin Ebadi, Roya Ahmadi, Vahid Amani, Hamid Reza Khavasi

**Affiliations:** a Islamic Azad University, Shahr-e-Rey Branch, Tehran, Iran; b Department of Chemistry, Islamic Azad University, Kazeroon Branch, Kazeroon, Fars, Iran; c Department of Chemistry, Shahid Beheshti University, Tehran 1983963113, Iran

## Abstract

The asymmetric unit of the title compound, [Hg_2_Br_4_(C_12_H_12_N_2_)_2_], contains one half-mol­ecule. The Hg^II^ atom is five-coordinated in a trigonal–bipyramidal configuration by two N atoms from the chelating 4,4′-dimethyl-2,2′-bipyridine ligand, two bridging Br and one terminal Br atom, leading to a centrosymmetric dimeric mol­ecule. There is a π–π contact between the pyridine rings [centroid-to-centroid distance = 3.756 (5) Å].

## Related literature

For related literature, see: Ahmadi, Kalateh, Ebadi *et al.* (2008 ▶); Ahmadi, Khalighi *et al.* (2008 ▶); Ahmadi, Kalateh, Abedi *et al.* (2008 ▶); Kalateh *et al.* (2008 ▶); Khalighi *et al.* (2008 ▶); Khavasi *et al.* (2008 ▶); Tadayon Pour *et al.* (2008 ▶); Yousefi, Rashidi Vahid *et al.* (2008 ▶); Yousefi, Tadayon Pour *et al.* (2008 ▶); Yousefi, Khalighi *et al.* (2008 ▶). For related structures, see: Craig *et al.* (1974 ▶); Perlepes *et al.* (1995 ▶).
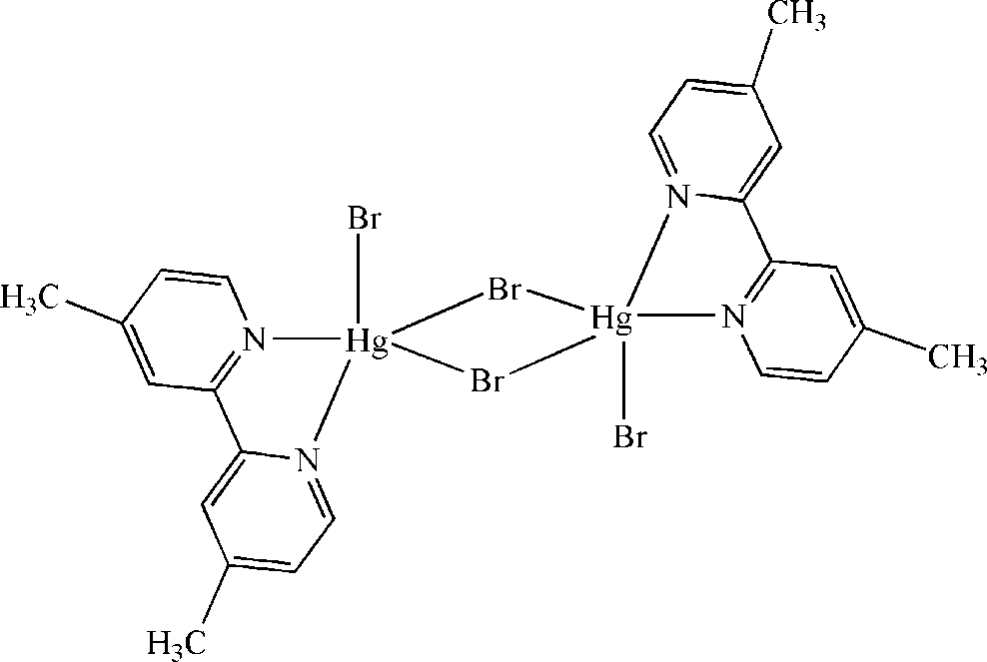


## Experimental

### 

#### Crystal data

[Hg_2_Br_4_(C_12_H_12_N_2_)_2_]
*M* *_r_* = 1089.25
Triclinic, 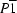

*a* = 7.3187 (15) Å
*b* = 9.2647 (19) Å
*c* = 11.345 (2) Å
α = 103.50 (3)°
β = 102.02 (3)°
γ = 107.87 (3)°
*V* = 678.6 (3) Å^3^
*Z* = 1
Mo *K*α radiation
μ = 17.21 mm^−1^
*T* = 120 (2) K
0.45 × 0.25 × 0.10 mm

#### Data collection

Bruker SMART CCD area-detector diffractometer
Absorption correction: numerical; shape of crystal determined optically (*X-SHAPE* and *X-RED*; Stoe & Cie, 2005 ▶) *T*_min_ = 0.008, *T*_max_ = 0.180
8289 measured reflections
3632 independent reflections
3504 reflections with *I* > 2σ(*I*)
*R*_int_ = 0.073

#### Refinement

*R*[*F* ^2^ > 2σ(*F* ^2^)] = 0.043
*wR*(*F* ^2^) = 0.165
*S* = 1.07
3632 reflections
155 parameters
H-atom parameters constrained
Δρ_max_ = 2.11 e Å^−3^
Δρ_min_ = −1.85 e Å^−3^

### 

Data collection: *SMART* (Bruker, 1998 ▶); cell refinement: *SAINT* (Bruker, 1998 ▶); data reduction: *SAINT*; program(s) used to solve structure: *SHELXTL* (Sheldrick, 2008 ▶); program(s) used to refine structure: *SHELXTL*; molecular graphics: *ORTEP-3 for Windows* (Farrugia, 1997 ▶); software used to prepare material for publication: *WinGX* (Farrugia, 1999 ▶).

## Supplementary Material

Crystal structure: contains datablocks I, global. DOI: 10.1107/S1600536808032510/hk2544sup1.cif

Structure factors: contains datablocks I. DOI: 10.1107/S1600536808032510/hk2544Isup2.hkl

Additional supplementary materials:  crystallographic information; 3D view; checkCIF report

## Figures and Tables

**Table 1:** Selected bond lengths (Å)

Br1—Hg1	2.5645 (15)
Br2—Hg1	2.7331 (11)
Br2—Hg1^i^	2.7884 (11)
N1—Hg1	2.409 (7)
N2—Hg1	2.346 (6)

Symmetry code: (i) 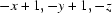.

**Fig. 1.**
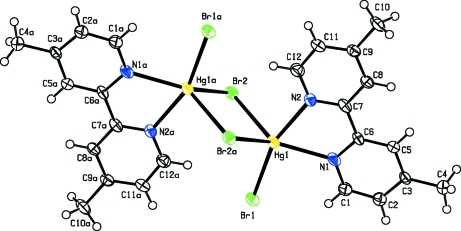
The molecular structure of the title molecule, with the atom-numbering scheme. Displacement ellipsoids are drawn at the 50% probability level.

**Fig. 2.**
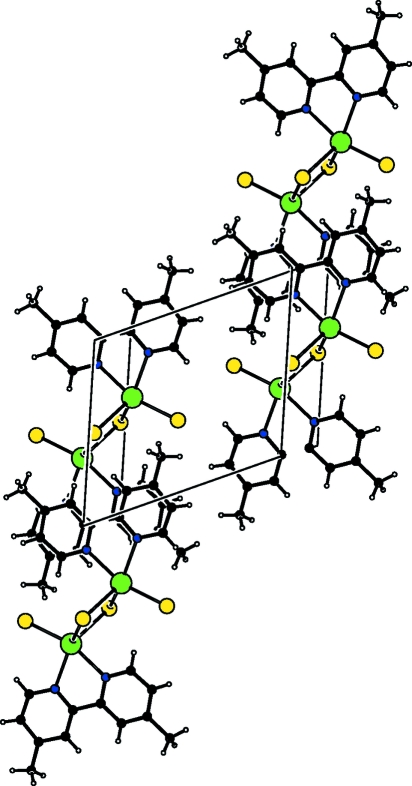
A partial packing diagram of the title compound.

## supplementary crystallographic information

### Comment

Recently, we reported the syntheses and crystal structures of [Zn(5,5'-dmbpy)Cl_2_], (II), (Khalighi *et al.*, 2008), [Zn(6-mbpy)Cl_2_], (III), (Ahmadi, Kalateh, Ebadi *et al.*, 2008), [HgI_2_(4,4'-dmbpy)], (IV), (Yousefi, Tadayon Pour *et al.*, 2008), [Cd(5,5'-dmbpy)(µ-Cl)_2_]_n_, (V), (Ahmadi, Khalighi *et al.*, 2008), [Hg(5,5'-dmbpy)I_2_], (VI), (Tadayon Pour *et al.*, 2008), [Cu(5,5'-dcbpy)(en)(H_2_O)_2_].2.5H_2_O, (VII), (Yousefi, Khalighi *et al.*, 2008), [Hg(dmphen)I_2_], (VIII), (Yousefi, Rashidi Vahid *et al.*, 2008), [In(4,4'-dmbpy)Cl_3_(DMSO)], (IX), (Ahmadi, Kalateh, Abedi *et al.*, 2008), [In(5,5'-dmbpy)Cl_3_(MeOH)], (X), (Kalateh *et al.*, 2008) and {[HgCl(dm4bt)]_2_(µ-Cl)_2_}, (XI), (Khavasi *et al.*, 2008) [where 5,5'-dmbpy is 5,5'-dimethyl-2,2'-bipyridine, 6-mbpy is 6-methyl-2,2'-bipyridine, 4,4'-dmbpy is 4,4'-dimethyl-2,2'-bipyridine, 5,5'-dcbpy is 2,2'-bipyridine-5,5'-dicarboxylate, en is ethylenediamine, dmphen is 4,7-diphenyl-1,10-phenanthroline, DMSO is dimethyl sulfoxide and dm4bt is 2,2'-dimethyl-4,4'-bithiazole]. There are two Hg^II^ dimer complexes, with formula, [{HgBr(N—N)}_2_(µ-Br)_2_], such as [{HgBr(bipy)}_2_(µ-Br)_2_], (XII), (Craig *et al.*, 1974) and [{HgBr(pquin)}_2_(µ-Br)_2_], (XIII), (Perlepes *et al.*, 1995) [where bipy is 2,2'-bipyridine and pquin is 2-(2'-pyridyl)quinoxaline] have been synthesized and characterized by single-crystal X-ray diffraction methods. We report herein the synthesis and crystal structure of the title compound, (I).

The asymmetric unit of the title compound, (I), contains one half-molecule (Fig. 1). The Hg^II^ atom is five-coordinated in a trigonal–bipyramidal configuration by two N atoms from the chelating 4,4'-dimethyl-2,2'-bipyridine ligand, two bridging Br and one terminal Br atoms. The Hg—Br and Hg—N bond lengths and angles (Table 1) are within normal ranges, as in (XII) and (XIII).

In the crystal structure, the π–π contact (Fig. 2) between the pyridine rings, Cg3–Cg4^i^ [symmetry code: (i) 2 - x, 2 - y, -z, where Cg3 and Cg4 are centroids of the rings (N1/C1–C3/C5–C6) and (N2/C7–C9/C11–C12), respectively] may stabilize the structure, with centroid–centroid distance of 3.756 (5) Å.

### Experimental

For the preparation of the title compound, (I), a solution of 4,4'-dimethyl-2,2'-bipyridine (0.20 g, 1.10 mmol) in methanol (5 ml) was added to a solution of HgBr_2_ (0.40 g, 1.10 mmol) in methanol (5 ml) at room temperature. The suitable crystals for X-ray analysis were obtained by methanol diffusion to a colorless solution in DMSO. Suitable crystals were isolated after one week (yield; 0.44 g, 73.4%).

### Refinement

H atoms were positioned geometrically, with C—H = 0.93 and 0.96 Å for aromatic and methyl H, respectively, and constrained to ride on their parent atoms with U_iso_(H) = 1.2U_eq_(C).

### Crystal data

**Table:**

[Hg_2_Br_4_(C_12_H_12_N_2_)_2_]	*Z* = 1
*M**_r_* = 1089.25	*F*(000) = 496
Triclinic, *P*1	*D*_x_ = 2.665 Mg m^−^^3^
Hall symbol: -P 1	Mo *K*α radiation, λ = 0.71073 Å
*a* = 7.3187 (15) Å	Cell parameters from 1005 reflections
*b* = 9.2647 (19) Å	θ = 1.9–29.2°
*c* = 11.345 (2) Å	µ = 17.21 mm^−^^1^
α = 103.50 (3)°	*T* = 120 K
β = 102.02 (3)°	Block, colourless
γ = 107.87 (3)°	0.45 × 0.25 × 0.10 mm
*V* = 678.6 (3) Å^3^	

### Data collection

**Table:**

Bruker SMART CCD area-detector diffractometer	3632 independent reflections
Radiation source: fine-focus sealed tube	3504 reflections with *I* > 2σ(*I*)
graphite	*R*_int_ = 0.073
φ and ω scans	θ_max_ = 29.2°, θ_min_ = 1.9°
Absorption correction: numerical shape of crystal determined optically (X-SHAPE and X-RED; Stoe & Cie, 2005)	*h* = −9→10
*T*_min_ = 0.008, *T*_max_ = 0.180	*k* = −12→12
8289 measured reflections	*l* = −15→15

### Refinement

**Table:**

Refinement on *F*^2^	Secondary atom site location: difference Fourier map
Least-squares matrix: full	Hydrogen site location: inferred from neighbouring sites
*R*[*F*^2^ > 2σ(*F*^2^)] = 0.043	H-atom parameters constrained
*wR*(*F*^2^) = 0.165	*w* = 1/[σ^2^(*F*_o_^2^) + (0.2*P*)^2^] where *P* = (*F*_o_^2^ + 2*F*_c_^2^)/3
*S* = 1.07	(Δ/σ)_max_ = 0.037
3632 reflections	Δρ_max_ = 2.11 e Å^−^^3^
155 parameters	Δρ_min_ = −1.85 e Å^−^^3^
0 restraints	Extinction correction: SHELXTL (Sheldrick, 1998), Fc^*^=kFc[1+0.001xFc^2^λ^3^/sin(2θ)]^-1/4^
Primary atom site location: structure-invariant direct methods	Extinction coefficient: 0.025 (3)

### Special details

**Table:**

Geometry. All e.s.d.'s (except the e.s.d. in the dihedral angle between two l.s. planes) are estimated using the full covariance matrix. The cell e.s.d.'s are taken into account individually in the estimation of e.s.d.'s in distances, angles and torsion angles; correlations between e.s.d.'s in cell parameters are only used when they are defined by crystal symmetry. An approximate (isotropic) treatment of cell e.s.d.'s is used for estimating e.s.d.'s involving l.s. planes.
Refinement. Refinement of *F*^2^ against ALL reflections. The weighted *R*-factor *wR* and goodness of fit *S* are based on *F*^2^, conventional *R*-factors *R* are based on *F*, with *F* set to zero for negative *F*^2^. The threshold expression of *F*^2^ > σ(*F*^2^) is used only for calculating *R*-factors(gt) *etc*. and is not relevant to the choice of reflections for refinement. *R*-factors based on *F*^2^ are statistically about twice as large as those based on *F*, and *R*- factors based on ALL data will be even larger.

### Fractional atomic coordinates and isotropic or equivalent isotropic displacement parameters (Å^2^)

**Table:**

	*x*	*y*	*z*	*U*_iso_*/*U*_eq_	
Hg1	0.53006 (3)	0.61812 (3)	−0.12572 (2)	0.0245 (2)	
Br1	0.40249 (13)	0.40548 (9)	−0.34549 (8)	0.0325 (2)	
Br2	0.23799 (9)	0.50603 (8)	−0.01775 (7)	0.0255 (2)	
N1	0.6268 (10)	0.8331 (7)	−0.2132 (6)	0.0274 (12)	
N2	0.6826 (10)	0.8668 (7)	0.0356 (7)	0.0259 (13)	
C1	0.5951 (13)	0.8128 (9)	−0.3382 (7)	0.0315 (15)	
H1	0.5267	0.7096	−0.3951	0.038*	
C2	0.6580 (13)	0.9356 (10)	−0.3863 (7)	0.0293 (15)	
H2	0.6341	0.9144	−0.4735	0.035*	
C3	0.7570 (11)	1.0912 (8)	−0.3051 (7)	0.0241 (12)	
C4	0.8350 (13)	1.2322 (10)	−0.3489 (8)	0.0309 (15)	
H4A	0.9788	1.2815	−0.3126	0.037*	
H4B	0.7755	1.3082	−0.3221	0.037*	
H4C	0.8009	1.1972	−0.4399	0.037*	
C5	0.7909 (11)	1.1122 (9)	−0.1742 (7)	0.0242 (13)	
H5	0.8591	1.2142	−0.1154	0.029*	
C6	0.7238 (10)	0.9826 (8)	−0.1321 (7)	0.0228 (12)	
C7	0.7541 (9)	0.9998 (8)	0.0047 (7)	0.0224 (12)	
C8	0.8549 (10)	1.1479 (8)	0.0993 (7)	0.0237 (13)	
H8	0.9095	1.2390	0.0774	0.028*	
C9	0.8745 (11)	1.1602 (8)	0.2269 (7)	0.0252 (13)	
C10	0.9781 (15)	1.3215 (9)	0.3311 (9)	0.0369 (18)	
H10A	0.9088	1.3911	0.3153	0.044*	
H10B	1.1152	1.3682	0.3319	0.044*	
H10C	0.9757	1.3071	0.4119	0.044*	
C11	0.8008 (11)	1.0223 (8)	0.2559 (7)	0.0272 (14)	
H11	0.8158	1.0256	0.3400	0.033*	
C12	0.7042 (12)	0.8786 (9)	0.1588 (9)	0.0300 (14)	
H12	0.6516	0.7860	0.1792	0.036*	

### Atomic displacement parameters (Å^2^)

**Table:**

	*U*^11^	*U*^22^	*U*^33^	*U*^12^	*U*^13^	*U*^23^
Hg1	0.0272 (3)	0.0176 (2)	0.0256 (3)	0.00731 (15)	0.00742 (15)	0.00320 (15)
Br1	0.0424 (4)	0.0215 (4)	0.0268 (4)	0.0077 (3)	0.0097 (3)	0.0018 (3)
Br2	0.0214 (4)	0.0225 (4)	0.0329 (4)	0.0098 (3)	0.0094 (3)	0.0064 (3)
N1	0.032 (3)	0.018 (3)	0.021 (3)	0.005 (2)	0.001 (2)	−0.002 (2)
N2	0.028 (3)	0.019 (2)	0.028 (3)	0.006 (2)	0.010 (2)	0.005 (2)
C1	0.043 (4)	0.023 (3)	0.019 (3)	0.006 (3)	0.005 (3)	0.002 (2)
C2	0.036 (4)	0.026 (4)	0.025 (3)	0.011 (3)	0.009 (3)	0.007 (3)
C3	0.026 (3)	0.016 (3)	0.031 (3)	0.010 (2)	0.011 (3)	0.005 (2)
C4	0.037 (4)	0.025 (3)	0.031 (4)	0.009 (3)	0.013 (3)	0.010 (3)
C5	0.029 (3)	0.021 (3)	0.023 (3)	0.010 (2)	0.010 (3)	0.005 (3)
C6	0.025 (3)	0.014 (3)	0.024 (3)	0.007 (2)	0.002 (2)	0.002 (2)
C7	0.016 (2)	0.018 (3)	0.024 (3)	0.006 (2)	−0.003 (2)	−0.001 (2)
C8	0.023 (3)	0.020 (3)	0.021 (3)	0.007 (2)	0.002 (2)	0.001 (2)
C9	0.030 (3)	0.025 (3)	0.023 (3)	0.015 (2)	0.007 (2)	0.005 (2)
C10	0.052 (5)	0.021 (3)	0.033 (4)	0.017 (3)	0.007 (3)	0.000 (3)
C11	0.028 (3)	0.024 (3)	0.025 (3)	0.012 (3)	0.005 (3)	0.001 (3)
C12	0.030 (3)	0.025 (3)	0.030 (4)	0.008 (3)	0.009 (3)	0.004 (3)

### Geometric parameters (Å, °)

**Table:**

Hg1—Br2^i^	2.7884 (11)	C5—H5	0.9300
Br1—Hg1	2.5645 (15)	C6—N1	1.341 (8)
Br2—Hg1	2.7331 (11)	C6—C7	1.484 (10)
Br2—Hg1^i^	2.7884 (11)	C7—N2	1.341 (10)
N1—Hg1	2.409 (7)	C7—C8	1.393 (9)
N2—Hg1	2.346 (6)	C8—C9	1.399 (10)
C1—C2	1.370 (11)	C8—H8	0.9300
C1—N1	1.347 (10)	C9—C11	1.371 (10)
C1—H1	0.9300	C9—C10	1.520 (10)
C2—C3	1.383 (10)	C10—H10A	0.9600
C2—H2	0.9300	C10—H10B	0.9600
C3—C5	1.410 (11)	C10—H10C	0.9600
C3—C4	1.497 (10)	C11—C12	1.377 (10)
C4—H4A	0.9600	C11—H11	0.9300
C4—H4B	0.9600	C12—N2	1.348 (11)
C4—H4C	0.9600	C12—H12	0.9300
C5—C6	1.383 (10)		
			
Br1—Hg1—Br2	102.48 (4)	H4A—C4—H4C	109.5
Br1—Hg1—Br2^i^	101.32 (4)	H4B—C4—H4C	109.5
Br2—Hg1—Br2^i^	87.29 (3)	C6—C5—C3	120.7 (7)
N1—Hg1—Br1	92.18 (15)	C6—C5—H5	119.9
N1—Hg1—Br2	135.51 (16)	C3—C5—H5	119.4
N1—Hg1—Br2^i^	130.97 (16)	N1—C6—C5	121.7 (7)
N2—Hg1—Br1	161.0 (2)	N1—C6—C7	115.9 (6)
N2—Hg1—Br2	93.17 (18)	C5—C6—C7	122.4 (6)
N2—Hg1—Br2^i^	89.92 (18)	N2—C7—C8	120.3 (7)
N2—Hg1—N1	69.0 (2)	N2—C7—C6	117.8 (6)
Hg1—Br2—Hg1^i^	92.71 (3)	C8—C7—C6	121.9 (6)
C1—N1—Hg1	124.3 (5)	C7—C8—C9	120.3 (7)
C6—N1—Hg1	118.0 (5)	C7—C8—H8	119.8
C6—N1—C1	117.6 (7)	C9—C8—H8	119.9
C7—N2—Hg1	119.1 (5)	C11—C9—C8	118.1 (7)
C12—N2—Hg1	121.6 (5)	C11—C9—C10	120.8 (7)
C12—N2—C7	119.3 (6)	C8—C9—C10	121.1 (7)
C2—C1—N1	123.7 (7)	C9—C10—H10A	109.4
C2—C1—H1	117.9	C9—C10—H10B	109.3
N1—C1—H1	118.4	H10A—C10—H10B	109.5
C1—C2—C3	120.0 (7)	C9—C10—H10C	109.7
C1—C2—H2	120.2	H10A—C10—H10C	109.5
C3—C2—H2	119.8	H10B—C10—H10C	109.5
C2—C3—C5	116.2 (7)	C9—C11—C12	119.2 (7)
C2—C3—C4	123.6 (7)	C9—C11—H11	120.9
C5—C3—C4	120.1 (6)	C12—C11—H11	119.9
C3—C4—H4A	109.7	N2—C12—C11	122.7 (7)
C3—C4—H4B	109.0	N2—C12—H12	118.6
H4A—C4—H4B	109.5	C11—C12—H12	118.7
C3—C4—H4C	109.7		
			
Hg1^i^—Br2—Hg1—Br1	101.00 (4)	C3—C5—C6—N1	0.8 (11)
Hg1^i^—Br2—Hg1—Br2^i^	0.0	C3—C5—C6—C7	−179.2 (6)
Hg1^i^—Br2—Hg1—N1	−152.7 (2)	N1—C6—C7—N2	−0.2 (9)
Hg1^i^—Br2—Hg1—N2	−89.77 (18)	C5—C6—C7—N2	179.7 (7)
C1—N1—Hg1—Br1	3.2 (7)	N1—C6—C7—C8	179.1 (6)
C6—N1—Hg1—Br1	−174.7 (5)	C5—C6—C7—C8	−0.9 (10)
C1—N1—Hg1—Br2	−107.1 (6)	N2—C7—C8—C9	−2.9 (10)
C1—N1—Hg1—Br2^i^	110.3 (6)	C6—C7—C8—C9	177.8 (6)
C6—N1—Hg1—Br2	74.9 (6)	C7—C8—C9—C11	3.2 (10)
C6—N1—Hg1—Br2^i^	−67.7 (6)	C7—C8—C9—C10	−178.0 (6)
C6—N1—Hg1—N2	2.7 (5)	C8—C9—C11—C12	−2.5 (10)
C1—N1—Hg1—N2	−179.3 (7)	C10—C9—C11—C12	178.7 (7)
C7—N2—Hg1—Br1	4.9 (9)	C9—C11—C12—N2	1.6 (11)
C12—N2—Hg1—Br1	−173.5 (4)	C5—C6—N1—C1	−0.4 (11)
C7—N2—Hg1—Br2	−140.9 (5)	C7—C6—N1—C1	179.5 (6)
C7—N2—Hg1—Br2^i^	131.8 (5)	C5—C6—N1—Hg1	177.7 (5)
C12—N2—Hg1—Br2	40.6 (6)	C7—C6—N1—Hg1	−2.4 (8)
C12—N2—Hg1—Br2^i^	−46.7 (6)	C2—C1—N1—C6	0.6 (13)
C7—N2—Hg1—N1	−2.9 (5)	C2—C1—N1—Hg1	−177.3 (7)
C12—N2—Hg1—N1	178.7 (6)	C11—C12—N2—C7	−1.3 (11)
N1—C1—C2—C3	−1.2 (13)	C11—C12—N2—Hg1	177.1 (5)
C1—C2—C3—C5	1.4 (12)	C8—C7—N2—C12	1.9 (10)
C1—C2—C3—C4	178.6 (8)	C6—C7—N2—C12	−178.7 (6)
C2—C3—C5—C6	−1.2 (11)	C8—C7—N2—Hg1	−176.5 (5)
C4—C3—C5—C6	−178.5 (7)	C6—C7—N2—Hg1	2.8 (8)

Symmetry codes: (i) −*x*+1, −*y*+1, −*z*.
